# A Cost-Effective Vehicle Localization Solution Using an Interacting Multiple Model−Unscented Kalman Filters (IMM-UKF) Algorithm and Grey Neural Network

**DOI:** 10.3390/s17061431

**Published:** 2017-06-18

**Authors:** Qimin Xu, Xu Li, Ching-Yao Chan

**Affiliations:** 1 School of Instrument Science and Engineering, Southeast University, Nanjing 210096, China; jimmy.xqm@gmail.com; 2 California Partners for Advanced Transportation Technology (PATH), University of California, Berkeley, CA 94720, USA; cychan@berkeley.edu

**Keywords:** vehicle localization, uncertain noise, Interacting Multiple Model, Grey Neural Network

## Abstract

In this paper, we propose a cost-effective localization solution for land vehicles, which can simultaneously adapt to the uncertain noise of inertial sensors and bridge Global Positioning System (GPS) outages. First, three Unscented Kalman filters (UKFs) with different noise covariances are introduced into the framework of Interacting Multiple Model (IMM) algorithm to form the proposed IMM-based UKF, termed as IMM-UKF. The IMM algorithm can provide a soft switching among the three UKFs and therefore adapt to different noise characteristics. Further, two IMM-UKFs are executed in parallel when GPS is available. One fuses the information of low-cost GPS, in-vehicle sensors, and micro electromechanical system (MEMS)-based reduced inertial sensor systems (RISS), while the other fuses only in-vehicle sensors and MEMS-RISS. The differences between the state vectors of the two IMM-UKFs are considered as training data of a Grey Neural Network (GNN) module, which is known for its high prediction accuracy with a limited amount of samples. The GNN module can predict and compensate position errors when GPS signals are blocked. To verify the feasibility and effectiveness of the proposed solution, road-test experiments with various driving scenarios were performed. The experimental results indicate that the proposed solution outperforms all the compared methods.

## 1. Introduction

Accurate and reliable vehicle ego-position is important and necessary information in more and more Intelligent Transportation System (ITS) applications [1,2,3]. The most popular technique is Global Positioning System (GPS), which can provide satisfactory localization performance in open areas [4,5], but in modern urban environments, more and more tall buildings or overpasses may affect the GPS signals and cause the failure of GPS. To improve the GPS localization performance, it is usually integrated with Inertial Navigation System (INS), which is a self-contained system and is not affected by external disturbances [4]. For land vehicles, the intent is to have a low-cost localization system [6,7], so in general only low-cost inertial sensors based on microelectromechanical systems (MEMS) are affordable enough [8,9]. In order to further lower the cost of vehicle localization systems, research efforts have recently been made to investigate the applicability of reduced inertial sensor systems (RISS) [10,11]. Usually, RISS involves a single-axis gyroscope and two-axis accelerometers. In RISS mechanization, pitch and roll are calculated from accelerometers instead of gyroscopes, and vehicle velocity is calculated from the forward speed derived from wheel speed sensors instead of accelerometers.

Although MEMS-based inertial sensors are portable and low-cost, their measurements often suffer from large and uncertain noises [8], which can seriously affect the localization performance. Unscented Kalman filter (UKF) has been widely used to fuse GPS and inertial sensor data in vehicle localization problems [12,13]. It can essentially provide derivative-free high order approximations of nonlinear models [14,15]. However, one of the limitations of UKF is the necessity to have priori statistical information of the process and measurement noises [16]. They are specified in the form of process noise covariances and measurement noise covariances in the filtering algorithm, normally expressed as the **Q** matrix and **R** matrix, respectively. Usually, the specification of the **R** matrix can be directly derived from the accuracy characteristics of the measurement devices, while the specification of the **Q** matrix is often determined by a trial-and-error approach and considered as a constant [17]. In a number of practical situations, due to the high uncertainty of the MEMS inertial sensor noise, the covariances are variable and difficult to determine. An improper specification will degrade the performance of the filter or even cause divergence. The adaptive filter algorithm has been considered as one of the strategies to adjust the covariance matrices through scale factors [18,19]. However, the approaches for determining the scale factors heavily rely on personal experience or computer simulation [20]. Besides, even small changes of the scale factor can greatly affect the final performance of the filter.

Alternatively, a structural adaptation approach called Interacting Multiple Model (IMM) makes it possible for a set of models with different characteristics to run in parallel [21,22]. IMM algorithms have already been applied to vehicle localization, and are usually used to represent the possible vehicle driving patterns with a set of models, which are generally established according to different maneuvering or driving conditions [21,22,23]. The IMM algorithm has shown better results than conventional switching schemes, because a smooth transition from one model to another is achieved [24]. Different from the common applications, we envisioned that the IMM algorithm can provide a soft switching among the filters designed for different noise levels and contribute to adapt to the uncertain noise of MEMS inertial sensors. To the authors’ knowledge, this application has seldom been evaluated or discussed in the existing literature.

Besides, in order to further compensate the position errors during GPS outages, artificial intelligence (AI) methods have attracted researchers’ interest due to their abilities of modeling and predicting for nonlinear system [25]. Usually, AI methods are used to model position errors when GPS signal is available and predict position errors during GPS outages. These AI-based approaches include Back-Propagation Neural Network (BPNN) [26], Support Vector Machine [27], etc. These approaches generally require a large number of training samples to achieve good generalization performance. However, to meet the real-time requirement of vehicle localization, a sliding window with a certain size is usually adopted to select training samples [28,29]. When there is a limited amount of samples, these approaches have a high probability of being affected [30]. 

Grey system theory can make full use of the historical data sequence information and is characterized by modeling with insufficient data [31]. However, grey system models have some drawbacks due to the lack of feedback, self-learning, and self-adaption. BPNN is one of the most popular learning algorithms and it can approximate an arbitrary nonlinear function with satisfactory precision [32]. Through mapping a grey system model to a BPNN, we can get a grey neural network (GNN) [33], which sufficiently exploits the advantages of both grey system theory and BPNN. We also develop a GNN module to model and predict position errors in this paper. To the authors’ knowledge, GNN is applied to vehicle localization for the first time.

This paper presents a cost-effective localization solution to adapt to uncertain inertial sensor noise and compensate position errors during GPS outages. The proposed vehicle localization solution integrates low-cost GPS, MEMS-RISS, and in-vehicle sensors. Here, the in-vehicle sensors specifically refer to wheel speed sensors and steering angle sensors. A novel IMM-UKF algorithm is proposed by introducing several UKFs designed for different noise levels into the framework of an IMM algorithm. Two IMM-UKFs are utilized to work in parallel when GPS is available. One fuses all the sensors including GPS, in-vehicle sensors, and RISS, while the other only fuses those which are not affected by the GPS-denied environments. The differences between the state vectors of the two IMM-UKFs are considered as the output of training samples. Meanwhile, the measurements of inertial sensors are considered as the corresponding input. A GNN module is adopted to establish the model and thus the position errors can be predicted and compensated during GPS outages. The proposed localization solution has been extensively evaluated in road-test experiments.

The rest of the paper is structured as follows. In the next section, we briefly describe the overview of the proposed localization solution. Then, in Section 3, we explain the detailed implementation of IMM-UKF algorithm. The design of GNN module is presented in Section 4. Section 5 presents the setup, scenarios, and results of experimental validation. Finally, a conclusion is presented in Section 6.

## 2. Overview of the Proposed Solution 

**Figure 1:**
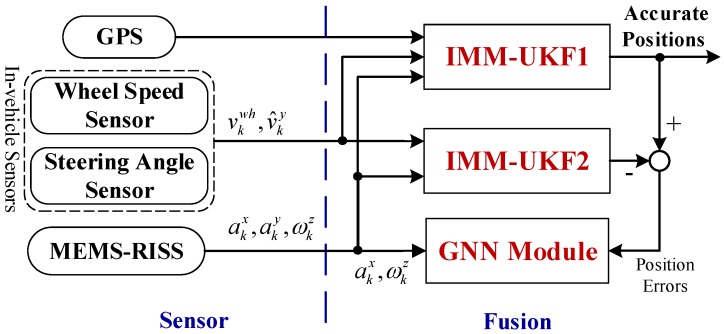
Whole diagram of the proposed localization solution.

The whole mechanism and functionality of the proposed localization solution is illustrated in Figure 1, which can be divided into two parts, i.e., the sensor part and the fusion part. In the sensor part, GPS, in-vehicle sensors, and MEMS-RISS are all cost-effective ones. For the MEMS-RISS, one single-axis gyroscope is mounted with its sensitive axis aligned with the vertical axis of the vehicle and measures the rotation rate of yaw angle in the body frame, denoted as $\omega_{k}^{z}$. Two accelerometers are mounted along the longitudinal and lateral axes of the vehicle, and the longitudinal and lateral accelerations of the vehicle are measured respectively, denoted as $a_{k}^{x}$ and $a_{k}^{y}$. For the in-vehicle sensors, since more and more vehicles are equipped with Antilock Brake System (ABS) and Electronic Stability Program (ESP), the information about vehicle forward speed and steering angle can be directly obtained from the controller area network (CAN) bus [22]. The wheel speed sensor can provide longitudinal velocity $v_{k}^{wh}$, while steering angle sensor can derive lateral velocity ${\hat{v}}_{k}^{y}$.

In the fusion part, improving the robustness against the uncertain noise and bridging the GPS outages are the main priorities. Specifically, the proposed IMM-UKF algorithm contains three UKFs designed for different noise characteristics and the output is a weighted sum of the three individual UKFs. The proposed algorithm can adapt to a wide variation of inertial sensor noise. When GPS is available, IMM-UKF1 fuses the information from GPS, in-vehicle sensors, and MEMS-RISS. Because GPS can provide direct position and velocity observations, more accurate vehicle positions can be achieved by IMM-UKF1. Meanwhile, IMM-UKF2 is introduced to work in parallel with IMM-UKF1. The IMM-UKF2 only fuse the information of in-vehicle sensors and MEMS-RISS. In order to bridge GPS outages, a GNN module is employed to establish the model of position errors. The difference between the state vectors of the two IMM-UKFs at each epoch is transferred to the GNN module as the desired output. While the RISS output is fed to the GNN as the corresponding input at the same epoch. Considering the balance between model accuracy and computation efficiency, a sliding window with a certain window size is considered for sample selection [27]. The GNN parameters are continuously updated till the occurrence of GPS outages to ensure the predicting precision.

**Figure 2:**
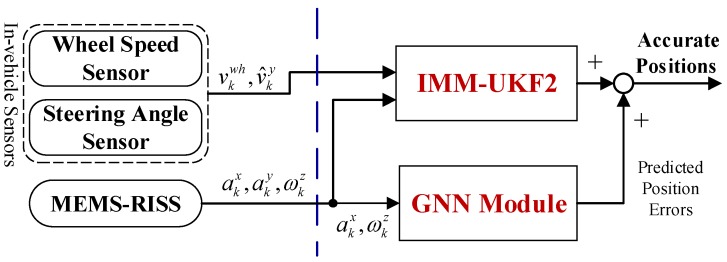
Diagram of the proposed localization solution operating without GPS.

When satellite signals are blocked, the absence of GPS observations causes the invalidation of IMM-UKF1 and it is removed from the system. However, the IMM-UKF2 can still efficiently execute the fusion of in-vehicle sensors and RISS. As shown in Figure 2, the GNN module can predict the position errors with the input of current RISS measurements. Due to the adaptation of IMM-UKF, even if the uncertain noise causes changes on the statistical properties, the proposed solution will not be affected and can still maintain the performance. Thus, accurate vehicle positions can be obtained even when the localization system suffers from GPS outages and uncertain noise of MEMS inertial sensors simultaneously.

## 3. Proposed IMM-UKF Algorithm

Since the inertial sensor noise is highly uncertain, a fixed value of noise statistics can lead to poor filter performance and even result in filter divergence. Thus, it is advisable to use IMM, which can represent the noise behavior with different characteristics and provide a soft switching among these noise characteristics. We study the IMM-UKF algorithm to adapt to the uncertain noise of inertial sensors. The details about IMM-UKF are shown in the following paragraphs.

### 3.1. Motion Model

The nonlinear motion model for the RISS involving attitude, velocity, and position states is presented in this section. When the vehicle is moving, the forward accelerometer measures the forward vehicle acceleration as well as the component due to gravity. Similarly, the transversal accelerometer measures the normal component of the vehicle acceleration as well as the component due to gravity. Thus, the pitch angle can be calculated by removing the vehicle acceleration derived from the wheel speed sensor measurements from the forward accelerometer measurements, while the roll angle can be calculated by compensating the transversal accelerometer measurements for the normal component of acceleration. The equations can be expressed as [34]:

(1) $$\begin{array}{c} p_{k}=\sin^{-1}\frac{a_{k}^{x}-{\dot{v}}_{k}^{wh}}{g}\\ r_{k}=-\sin^{-1}\frac{a_{k}^{y}+\omega_{k}^{z}v_{k}^{wh}}{g\cos p_{k}} \end{array}$$

where the subscript k represents the time step, $p_{k}$ and $r_{k}$ are pitch and roll angle, respectively.${\dot{v}}_{k}^{wh}$ is the differentiation of $v_{k}^{wh}$, g denotes the acceleration due to gravity.

Note that $v_{k}^{wh}$ is derived from the wheel speed sensor rather than the longitudinal accelerometer. This is because any uncompensated accelerometer bias will introduce an error to the speed during the integration. However, the speed derived from the wheel speed sensor avoids the integration. Besides, ${\dot{v}}_{k}^{wh}$ at each time step can be calculated as:

(2) $${\dot{v}}_{k}^{wh}=\frac{v_{k}^{wh}-v_{k-1}^{wh}}{dt}$$

where dt is the time interval between $v_{k-1}^{wh}$ and $v_{k}^{wh}$. Since the output frequency of vehicle speed is 100 Hz, dt is 0.01 s here.

When calculating the azimuth angle, both the Earth’s rotation and the change of orientation of the local-level frame are taken into consideration [35]. Thus, the calculation of azimuth angle $A_{k}$ is:

(3) $$A_{k}=\tan^{-1}\frac{U^{E}}{U^{N}}+\left(\omega^{e}\sin\varphi_{k-1}\right)\Delta t+\frac{v_{k-1}^{wh}\sin A_{k-1}\cos p_{k-1}\tan\varphi_{k-1}}{R_{N}+h_{k-1}}\Delta t$$

where:

$U^{E}=\sin A_{k-1}\cos p_{k-1}\cos\gamma_{k}^{z}-\left(\cos A_{k-1}\cos r_{k-1}+\sin A_{k-1}\sin p_{k-1}\sin r_{k-1}\right)\sin\gamma_{k}^{z}$
$U^{N}=\cos A_{k-1}\cos p_{k-1}\cos\gamma_{k}^{z}-\left(-\sin A_{k-1}\cos r_{k-1}+\cos A_{k-1}\sin p_{k-1}\sin r_{k-1}\right)\sin\gamma_{k}^{z}$
$\gamma_{k}^{z}=\omega_{k}^{z}\Delta t$, $\omega^{e}$ is the Earth’s rotation rate,
$\varphi_{k}$ is the latitude of the vehicle position,
$h_{k}$ is the altitude,
$R_{N}$ is the normal radius of curvature of the Earth.

Since the vehicle does not jump off the ground during common driving maneuvers [36], the vertical velocity can be presumed to be zero. Thus, the relationship between the vehicle’s velocity in the body frame and in the local-level frame (navigation frame) can be calculated as:

(4) $$\left[\begin{array}{c} v_{k}^{E}\\ v_{k}^{N}\\ v_{k}^{U} \end{array}\right]=R_{b,l}\left[\begin{array}{c} v_{k}^{x}\\ v_{k}^{y}\\ 0 \end{array}\right]$$

where $v_{k}^{E}$ is the velocity component along the east direction, $v_{k}^{N}$ is the velocity component along the north direction, $v_{k}^{U}$ is the velocity component along the up direction. $v_{k}^{x}$ is the velocity component along the forward longitudinal direction, and it can be calculated from the longitudinally aligned accelerometer as $v_{k}^{x}=v_{k-1}^{x}+a_{k}^{x}\Delta t$. $v_{k}^{y}$ is the velocity component along the transversal direction, and it can be calculated from the laterally aligned accelerometer as $v_{k}^{y}=v_{k-1}^{y}+a_{k}^{y}\Delta t$. $R_{b,l}$ is the rotation matrix that transforms from the vehicle body frame to the local-level frame, and is given as:

$$R_{b,l}=[\begin{array}{ccc} \sin A_{k}\cos p_{k} & \cos A_{k}\cos r_{k}+\sin A_{k}\sin p_{k}\sin r_{k} & \cos A_{k}\sin r_{k}-\sin A_{k}\sin p_{k}\cos r_{k}\\ \cos A_{k}\cos p_{k} & -\sin A_{k}\cos r_{k}+\cos A_{k}\sin p_{k}\sin r_{k} & -\sin A_{k}\sin r_{k}-\cos A_{k}\sin p_{k}\cos r_{k}\\ \sin p_{k}\; & -\cos p_{k}\sin r_{k} & \cos p_{k}\cos r_{k} \end{array}]$$

Expanding Equation (4), we can get:

(5) $$v_{k}^{E}=v_{k}^{x}\sin A_{k}\cos p_{k}+v_{k}^{y}\left(\cos A_{k}\cos r_{k}+\sin A_{k}\sin p_{k}\sin r_{k}\right)\;v_{k}^{N}=v_{k}^{x}\cos A_{k}\cos p_{k}+v_{k}^{y}\left(-\sin A_{k}\cos r_{k}+\cos A_{k}\sin p_{k}\sin r_{k}\right)\;v_{k}^{U}=v_{k}^{x}\sin p_{k}-v_{k}^{y}\cos p_{k}\sin r_{k}$$

Then, the position calculation can be expressed as:

(6) $$\begin{array}{c} \varphi_{k}=\varphi_{k-1}+\frac{v_{k}^{N}}{R_{M}+h_{k}}\Delta t\\ \lambda_{k}=\lambda_{k-1}+\frac{v_{k}^{E}}{\left(R_{N}+h_{k}\right)\cos\varphi_{k}}\Delta t\\ h_{k}=h_{k-1}+v_{k}^{U}\Delta t \end{array}$$

where $\lambda_{k}$ is the longitude of the vehicle position, $R_{M}$ is the meridian radius of curvature of the Earth. Based on Equations (1), (3), (5) and (6), the discrete-time system state equation can be presented as:

(7) $$X_{k}=f\left(X_{k-1},u_{k}\right)+W_{k}$$

where $X_{k}$ and $u_{k}$ represent the state vector and the input vector respectively, $W_{k}$ is the corresponding system noise vector, f(·) is the nonlinear system function. $X_{k}$, $u_{k}$, and f(·) can be described as:

(8) $$X_{k}=\left[\begin{array}{ccc} \begin{array}{ccc} \varphi_{k} & \lambda_{k} & h_{k} \end{array} & \begin{array}{ccc} v_{k}^{E} & v_{k}^{N} & v_{k}^{U} \end{array} & \begin{array}{ccc} p_{k} & r_{k} & A_{k} \end{array} \end{array}\right]\prime$$

(9) $$u_{k}=\left[\begin{array}{cc} \begin{array}{cc} v_{k}^{od} & a_{k}^{x} \end{array} & \begin{array}{cc} a_{k}^{y} & \omega_{k}^{z} \end{array} \end{array}\right]\prime$$

(10) $$f\left(\cdot \right)=[\begin{array}{c} \varphi_{k-1}+\frac{v_{k}^{N}}{R_{M}+h_{k}}\Delta t\\ \lambda_{k-1}+\frac{v_{k}^{E}}{\left(R_{N}+h_{k}\right)\cos\varphi_{k}}\Delta t\\ h_{k-1}+v_{k}^{U}\Delta t\\ v_{k}^{x}\sin A_{k}\cos p_{k}+v_{k}^{y}(\cos A_{k}\cos r_{k}+\sin A_{k}\sin p_{k}\sin r_{k}\\ v_{k}^{x}\cos A_{k}\cos p_{k}+v_{k}^{y}\left(-\sin A_{k}\cos r_{k}+\cos A_{k}\sin p_{k}\sin r_{k}\right)\\ v_{k}^{x}\sin p_{k}-v_{k}^{y}\cos p_{k}\sin r_{k}\\ \sin^{-1}\frac{a_{k}^{x}-{\dot{v}}_{k}^{x}}{g}\\ -\sin^{-1}\frac{a_{k}^{y}+\omega_{k}^{z}v_{k}^{x}}{g\cos p_{k}}\\ \tan^{-1}\left(\frac{U^{E}}{U^{N}}\right)+\left(\omega^{e}\sin\varphi_{k-1}\right)\Delta t+\frac{v_{k-1}^{E}\tan\varphi_{k-1}}{R_{N}+h_{k-1}}\Delta t \end{array}]$$

### 3.2. Observation Model

As shown in Figure 1, the observation information comes from two sources, i.e., the in-vehicle sensors and the GPS. The observation equation of the in-vehicle sensors can be established as:

(11) $$Z_{k}^{1}=h_{1}\left(X_{k}\right)+n_{1}=\left[\begin{array}{c} v_{k}^{E}\cos A_{k}+v_{k}^{N}\sin A_{k}+n_{vx}\\ -v_{k}^{E}\sin A_{k}+v_{k}^{N}\cos A_{k}+n_{vy} \end{array}\right]$$

where $Z_{k}^{1}=\left[\begin{array}{cc} v_{k}^{wh} & {\hat{v}}_{k}^{y} \end{array}\right]\prime$, ${\hat{v}}_{k}^{y}$ is the vehicle lateral velocity derived from front wheel steering angle data. $n_{1}=\left[\begin{array}{cc} n_{vx} & n_{vy} \end{array}\right]\prime$ is the corresponding observation noise vector.

In order to estimate the lateral velocity from front wheel steering angle data in real time, we adopt the simple but effective bicycle model [37,38]. Moreover, to achieve more accurate estimation, the influences of roll and pitch angles are also considered. Assume that inner tires and outer tires have the same tire cornering stiffnesses and tire slip angles, the equations for the lateral motion of the vehicle can be established according to Newton’s law of motion [34], described as:

(12) $$m\left({\dot{v}}_{k}^{y}+\omega_{k}^{z}v_{k}^{wh}\right)=2F_{k}^{sf}+2F_{k}^{sr}$$

where m is the mass of the vehicle. $F_{k}^{sf}$ and $F_{k}^{sr}$ are the front-tire lateral force and the rear-tire lateral force, respectively. The tire slip is usually small and the tire lateral forces $F_{sf}$ and $F_{sr}$ can usually be approximated by a linear function [22], expressed as:

(13) $$F_{k}^{sf}=C_{af}\alpha_{k}^{f},\;F_{k}^{sr}=C_{ar}\alpha_{k}^{r}$$

where $C_{af}$ and $C_{ar}$ are the front tire cornering stiffness and rear tire cornering stiffness, respectively. $\alpha_{k}^{f}$ and $\alpha_{k}^{r}$ are the front-tire slip angle and rear-tire slip angle respectively, and they can be described as:

(14) $$\alpha_{k}^{f}=\delta_{k}^{f}-\frac{{\hat{v}}_{k}^{y}-a\omega_{k}^{z}}{v_{k}^{wh}},\;\alpha_{k}^{r}=\frac{b\omega_{k}^{z}-{\hat{v}}_{k}^{y}}{v_{k}^{wh}}$$

where $\delta_{k}^{f}$ denotes the front-wheel steering angle, a and b are the distance between the center of gravity (CoG) and the front axle and the distance between the CoG and the rear axle, respectively.

Substituting Equations (13) and (14) into Equation (12), we can obtain:

(15) $${\dot{v}}_{k}^{y}=\frac{\beta_{1}}{v_{k}^{wh}}{\hat{v}}_{k}^{y}+\left(\frac{\beta_{2}}{v_{k}^{wh}}-v_{k}^{wh}\right)\omega_{k}^{z}+\beta_{3}\delta_{k}^{f}$$

where $\beta_{1}=\frac{-2\left(C_{af}+C_{ar}\right)}{m}$, $\beta_{2}=\frac{2\left(C_{ar}b-C_{ar}a\right)}{m}$, $\beta_{3}=\frac{2C_{af}}{m}$.

Thus, the ${\hat{v}}_{k}^{y}$ can be calculated as:

(16) $${\hat{v}}_{k}^{y}={\hat{v}}_{k-1}^{y}+{\dot{v}}_{k}^{y}\Delta t$$

Furthermore, the observation equation of the GPS measurements is:

(17) $$Z_{k}^{2}=h_{2}\left(X_{k}\right)+n_{2}=\left[\begin{array}{c} \varphi_{k}+n_{\varphi }\\ \lambda_{k}+n_{\lambda }\\ h_{k}+n_{h}\\ v_{k}^{E}+n_{vE}\\ v_{k}^{N}+n_{vN}\\ v_{k}^{U}+n_{vU} \end{array}\right]$$

where $Z_{k}^{2}=[\begin{array}{cccccc} \varphi_{k}^{G} & \lambda_{k}^{G} & h_{k}^{G} & v_{k}^{GE} & v_{k}^{GN} & v_{k}^{GU} \end{array}]\prime$. $\varphi_{k}^{G}$, $\lambda_{k}^{G}$, and $h_{k}^{G}$ are the latitude, longitude, and altitude output by GPS, respectively. $v_{k}^{GE}$, $v_{k}^{GN}$, and $v_{k}^{GU}$ are the east, north and up velocity measured by GPS, respectively. $n_{2}=[\begin{array}{cccccc} n_{\varphi } & n_{\lambda } & n_{h} & n_{vE} & n_{vN} & n_{vU} \end{array}]\prime$ is the corresponding observation noise vector.

### 3.3. Implementation of the Proposed Algorithm

**Figure 3:**
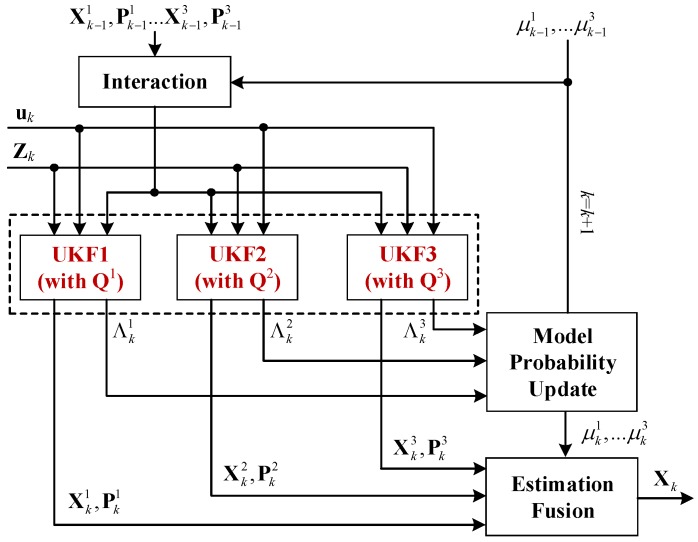
Proposed IMM-UKF algorithm.

In our study, the IMM-UKF approach contains three UKFs with different **Q** matrices, as shown in Figure 3. Using the system state equation and measurement equation described above, we can execute the recursive procedure of the proposed IMM-UKF algorithm, which can be described in four parts [22,23]:

(1) Interaction

The individual filter estimation $X_{k-1}^{i}$ of the *i*th UKF (*i* = 1,2,3) is mixed with the predicted model probability $\mu_{k-1}^{i}$ and the Markov transition probability $\pi_{ji}$, i.e., the probability of the transition from state *j* to state *i*, to give:

(18) $$\mu_{k,k-1}^{i}=\sum_{j=1}^{3}\pi_{ji}\mu_{k-1}^{j}(i=1,2,3)$$

The mixing weight is given by:

(19) $$\mu_{k-1}^{j|i}=\frac{\pi_{ji}\mu_{k-1}^{j}}{\mu_{k,k-1}^{i}}(i,j=1,2,3)$$

The mixing of the state estimates ${\bar{X}}_{k-1}^{i}$ can be computed as:

(20) $${\bar{X}}_{k-1}^{i}=\sum_{j=1}^{3}\mu_{k-1}^{j|i}X_{k-1}^{j}(i=1,2,3)$$

The mixing of the covariance ${\bar{P}}_{k-1}^{i}$ is given as:

(21) $${\bar{P}}_{k-1}^{i}=\sum_{j=1}^{3}\mu_{k-1}^{j|i}\left\{P_{k-1}^{j}+\left[{\bar{X}}_{k-1}^{i}-X_{k-1}^{j}\right]\left[{\bar{X}}_{k-1}^{i}-X_{k-1}^{j}\right]\prime \right\}(i=1,2,3)$$

(2) Specific Filtering

Using the mixing state and covariance obtained in the interaction step, each UKF predicts and updates the model state and covariance individually. Since the specification of Q matrix depends on the noise characteristics of inertial sensors [39], UKF1 is designed for high-level noise with Q^1^, UKF2 is designed for medium-level noise with Q^2^, and UKF3 is designed for low-level noise with Q^3^. The execution of the *i*th UKF (*i* = 1,2,3) can be described as follows:

Step 1: Calculate the Sigma Points

The Cholesky factorization is utilized in obtaining the sigma points, which is numerical efficient and stable, given by:

(22) $$\{\begin{array}{c} \xi_{k-1}^{iq}={\bar{X}}_{k-1}^{i}\;q=0\\ \xi_{k-1}^{iq}={\bar{X}}_{k-1}^{i}\;+\sqrt{\left(n+\eta \right)}{\left\{chol\left({\bar{P}}_{k-1}^{i}\right)\right\}}_{q}^{\prime }\;q=1,2,\ldots ,n\\ \xi_{k-1}^{iq}={\bar{X}}_{k-1}^{i}\;-\sqrt{\left(n+\eta \right)}{\left\{chol\left({\bar{P}}_{k-1}^{i}\right)\right\}}_{q-n}^{\prime }\;q=n+1,n+2,\ldots ,2n \end{array}$$

where n is the dimension of state vector X, $\eta =\alpha_{1}^{2}\left(n+\alpha_{2}\right)-n$ is a scaling parameter $\alpha_{1}$ determines the spread of the sigma points around ${\bar{X}}_{k-1}^{i}$ and is usually set to a small positive value. $\alpha_{2}$ is a secondly scaling parameter. $\left\{chol\left({\bar{P}}_{k-1}^{i}\right)\right\}\prime$ is the lower-triangular matrix of the Cholesky factorization of ${\bar{P}}_{k-1}^{i}$, the subscript q means the *q*th column.

Step 2: Time Propagation

(23) $$\xi_{k,k-1}^{iq}=f\left(\xi_{k-1}^{iq},u_{k-1}\right)\;q=0,1,\ldots ,2n$$

(24) $${\hat{X}}_{k,k-1}^{i}=\sum_{q=0}^{2n}\omega_{q}^{\left(m\right)}\xi_{k,k-1}^{iq}$$

(18) $$P_{k.k-1}^{i}=\overset{2n}{\underset{q=0}{\sum }}\omega_{q}^{\left(c\right)}\left[\xi_{k,k-1}^{iq}-{\hat{X}}_{k,k-1}^{i}\right]\cdot {\left[\xi_{k,k-1}^{iq}-{\hat{X}}_{k,k-1}^{i}\right]}^{\prime }+Q^{i}$$

(26) $$\zeta_{k,k-1}^{iq}=h\left(\xi_{k,k-1}^{iq}\right)\;q=0,1,\ldots ,2n$$

(27) $${\hat{Z}}_{k,k-1}^{i}=\overset{2n}{\underset{q=0}{\sum }}\omega_{q}^{\left(m\right)}\zeta_{k,k-1}^{iq}$$

where the weighting factors are calculated as:

$$\{\begin{array}{c} \omega_{0}^{\left(m\right)}=\frac{\eta }{n+\eta }\;\\ \omega_{0}^{\left(c\right)}=\frac{\eta }{n+\eta }+\left(1-{\alpha_{1}}^{2}+\alpha_{3}\right)\;\\ \omega_{q}^{\left(m\right)}=\omega_{q}^{\left(m\right)}=\frac{1}{2\left(n+\eta \right)}\;q=1,2,\ldots ,2n \end{array}$$

$\alpha_{3}$ is used to incorporate prior knowledge of the distribution of ${\bar{X}}_{k-1}^{i}$ and is optimally set to 2 for Gaussian distributions. Note that h(·) is the combination of $h_{1}\left(\cdot \right)$ and $h_{2}\left(\cdot \right)$ in IMM-UKF1, while it is equal to $h_{1}\left(\cdot \right)$ in IMM-UKF2.

Step 3: Measurement Update

(28) $$P_{ZZ}^{i}=\overset{2n}{\underset{q=0}{\sum }}\omega_{q}^{\left(c\right)}\left[\zeta_{k,k-1}^{iq}-{\hat{Z}}_{k,k-1}^{i}\right]\cdot {\left[\zeta_{k,k-1}^{iq}-{\hat{Z}}_{k,k-1}^{i}\right]}^{\prime }+R$$

(29) $$P_{XZ}^{i}=\overset{2n}{\underset{i=0}{\sum }}\omega_{q}^{\left(c\right)}\left[\xi_{k,k-1}^{iq}-{\hat{X}}_{k,k-1}^{i}\right]\cdot {\left[\zeta_{k,k-1}^{iq}-{\hat{Z}}_{k,k-1}^{i}\right]}^{\prime }$$

(30) $$K_{k}^{i}=P_{XZ}^{i}{\left(P_{ZZ}^{i}\right)}^{-1}$$

(31) $$X_{k}^{i}={\hat{X}}_{k,k-1}^{i}+K_{k}^{i}\left(Z_{k}-{\hat{Z}}_{k,k-1}^{i}\right)$$

(32) $$P_{k}^{i}=P_{k.k-1}^{i}-K_{k}^{i}P_{ZZ}^{i}{\left(K_{k}^{i}\right)}^{\prime }$$

Note that $Z_{k}$ is the combination of $Z_{k}^{1}$ and $Z_{k}^{2}$ in IMM-UKF1, while it is equal to $Z_{k}^{1}$ in IMM-UKF2.

(3) Model probability Update

Under Gaussian statistics assumption, the likelihood for the observation can be calculated from the innovation vector $v_{k}^{i}$ and its covariance $s_{k}^{i}$ as follows:

(33) $$\Lambda_{k}^{i}=\frac{\exp\left\{-\left(\frac{1}{2}\right){\left(v_{k}^{i}\right)}^{\prime }{\left(s_{k}^{i}\right)}^{-1}v_{k}^{i}\right\}}{\sqrt{\left|2\pi s_{k}^{i}\right|}}(i=1,2,3)$$

where $v_{k}^{i}=Z_{k}-{\hat{Z}}_{k,k-1}^{i}$, $s_{k}^{i}=P_{ZZ}^{i}$

Then, the model probability update is calculated as:

(34) $$\mu_{k}^{i}=\frac{\mu_{k,k-1}^{i}\Lambda_{k}^{i}}{\sum_{j=1}^{3}\mu_{k,k-1}^{j}\Lambda_{k}^{j}}(i=1,2,3)$$

(4) Estimation Fusion

Finally, the combined state $X_{k}$ can be calculated as:

(35) $$X_{k}=\sum_{i=1}^{3}\mu_{k}^{i}X_{k}^{i}$$

Since the proposed IMM-UKF can adapt to a wide variation of inertial sensor noise, the vehicle localization system is robust enough to achieve an accurate position output when facing uncertain inertial sensor noise.

## 4. Design the GNN Module

Considering the uncertain noise of MEMS inertial sensors and varied driving situations, it is very difficult to establish appropriate functions or equations to describe the dynamic behaviors of RISS position errors. The grey system theory requires only a limited amount of data to estimate the behavior of unknown systems. Through combing grey system theory with neural network, the predicting precision can be raised undoubtedly when the training samples are not sufficient. Therefore, the GNN module is developed here to predict the future position errors using the current available inertial sensor data.

For land vehicle applications, the horizontal localization performance is generally the main concern [7,40]. Thus, the latitude and longitude components of the difference between two state vectors associated with IMM-UKF1 and IMM-UKF2 are selected as the outputs. Since the vehicle maneuverability can affect the position errors [41], the longitudinal acceleration $a_{k}^{x}$ and the yaw rate $\omega_{k}^{z}$ measured by RISS are considered as the corresponding inputs. In actual implementation, two separate GNNs are designed in parallel for the position errors along latitude and longitude, respectively. It is worthwhile to mention here that the two GNNs have similar designing process. For simplicity, we choose the latitude component to show how to establish the GNN while the other one for the longitude component can be processed similarly. The GNN for the latitude component is developed as follows [42]:

(1) Construct the original data series:

(36) $$\begin{array}{c} z_{t}^{\left(0\right)}=X_{t}^{IMM-UKF1}\left[1\right]-X_{t}^{IMM-UKF2}\left[1\right]\\ y_{t}^{1\left(0\right)}=a_{t}^{x}\;\\ y_{t}^{2\left(0\right)}=\omega_{t}^{z}\; \end{array}t=1,2,\ldots ,N$$

where $X_{t}^{IMM-UKF1}\left[1\right]$ and $X_{t}^{IMM-UKF2}\left[1\right]$ are the latitude component of the state vector of IMM-UKF1 and IMM-UKF2 at time step t, respectively. N is the length of the sliding window and can be adjusted according to the length of the assumed GPS-outage time.

(2) Take accumulated generating operation (AGO) on $z_{t}^{\left(0\right)}$, $y_{t}^{1\left(0\right)}$, and $y_{t}^{2\left(0\right)}$, respectively. Then the AGO sequence can be obtained as:

(37) $$\begin{array}{c} Z^{\left(1\right)}=\left(z_{1}^{\left(1\right)},\;z_{2}^{\left(1\right)},\;\ldots ,\;z_{N}^{\left(1\right)}\right)\\ y^{1\left(1\right)}=\left(y_{1}^{1\left(1\right)},y_{2}^{1\left(1\right)},\ldots \ldots ,y_{N}^{1\left(1\right)}\right)\\ y^{2\left(1\right)}=\left(y_{1}^{2\left(1\right)},\;y_{2}^{2\left(1\right)},\;\ldots ,\;y_{N}^{2\left(1\right)}\right) \end{array}$$

where $z_{t}^{\left(1\right)}=\overset{t}{\underset{i=1}{\sum }}z_{i}^{\left(0\right)}$, $y_{t}^{1\left(1\right)}=\overset{t}{\underset{i=1}{\sum }}y_{i}^{1\left(0\right)}$, $y_{t}^{2\left(1\right)}=\overset{t}{\underset{i=1}{\sum }}y_{i}^{2\left(0\right)}$, t=1,2,…,N.

(3) Form the whitening differential equation according to grey system theory:

(38) $$\frac{dz_{t}^{\left(1\right)}}{dt}+b_{1}z_{t}^{\left(1\right)}=b_{2}y_{t}^{1\left(1\right)}+b_{3}y_{t}^{2\left(1\right)}$$

The solution of Equation (38) can be obtained as:

(39) $${\hat{z}}_{t}^{\left(1\right)}=\left(z_{1}^{\left(1\right)}-\frac{b_{2}}{b_{1}}y_{t}^{1\left(1\right)}-\frac{b_{3}}{b_{1}}y_{t}^{2\left(1\right)}\right)e^{-b_{1}t}+\frac{b_{2}}{b_{1}}y_{t}^{1\left(1\right)}+\frac{b_{3}}{b_{1}}y_{t}^{2\left(1\right)}$$

Let $d=\frac{b_{2}}{b_{1}}y_{t}^{1\left(1\right)}+\frac{b_{3}}{b_{1}}y_{t}^{2\left(1\right)}$, and Equation (39) can be transformed to:

(40) $$\;{\hat{z}}_{t}^{\left(1\right)}=\left(\left(z_{1}^{\left(1\right)}-d\right)\cdot \frac{e^{-b_{1}t}}{1+e^{-b_{1}t}}+d\cdot \frac{1}{1+e^{-b_{1}t}}\right)\cdot \left(1+e^{-b_{1}t}\right)=\left(\left(z_{1}^{\left(1\right)}-d\right)-z_{1}^{\left(1\right)}\cdot \frac{1}{1+e^{-b_{1}t}}+2d\cdot \frac{1}{1+e^{-b_{1}t}}\right)\cdot \left(1+e^{-b_{1}t}\right)$$

**Figure 4:**
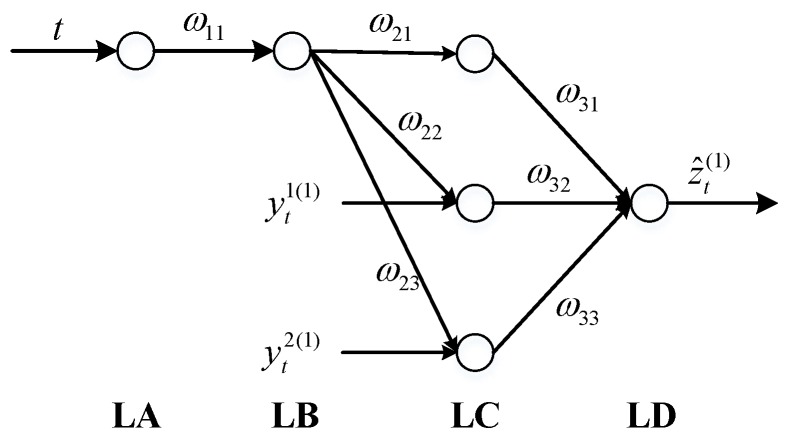
Schematic diagram of GNN for the application in this paper.

(4) Map Equation (40) to an expanded BPNN. Thus we can obtain the GNN with two input variables and one output variable, as shown in Figure 4.

Here, *t* is the time step and can also be treated as one hidden input. *ω*_11_, *ω*_21_, *ω*_22_, *ω*_23_, *ω*_31_, *ω*_32_, *ω*_33_ are network weighting values. LA, LB, LC, LD are the four layers of GNN respectively. The learning process of GNN is as follows:

*Step 1:* Initialize the network parameters and weighting values.

Let $\frac{2b_{2}}{b_{1}}=u_{1}$ and $\frac{2b_{3}}{b_{1}}=u_{2}$, then the network initial weighting value can be represented as: $\omega_{11}=b_{1}$, $\omega_{21}=-z_{1}^{\left(1\right)}$, $\omega_{21}=u_{1}$, $\omega_{23}=u_{2}$, $\omega_{31}=\omega_{32}=\omega_{33}=1+e^{-b_{1}t}$

*Step 2:* Calculate the output of each layer at each time step.

LA layer: $o_{a}=\omega_{11}t$
LB layer: $o_{b}=\frac{1}{1+e^{-\omega_{11}t}}$
LC layer: $o_{c1}=o_{b}\omega_{21}$, $o_{c2}=y_{t}^{1\left(1\right)}o_{b}\omega_{22}$, $o_{c3}=y_{t}^{2\left(1\right)}o_{b}\omega_{23}$
LD layer: $o_{d}=o_{c1}\omega_{31}+o_{c2}\omega_{32}+o_{c1}\omega_{33}-\theta$

where θ is the threshold value and can be calculated as $\theta =\left(1-e^{-b_{1}t}\right)\left(d-z_{1}^{\left(1\right)}\right)$.

*Step 3:* Calculate the errors between the forecast and expectation, and then adjust the weighting values and the threshold value.

The error of each level can be calculated as:

LD layer error: $\delta_{d}=o_{d}-z_{t}^{\left(1\right)}$

LC layer error: $\delta_{c1}=\delta_{c2}=\delta_{c3}=\delta_{d}\left(1+e^{-\omega_{11}t}\right)$

LB layer error: $\delta_{b}=\frac{1}{1+e^{-\omega_{11}t}}\left(1-\frac{1}{1+e^{-\omega_{11}t}}\right)\left(\omega_{21}\delta_{c1}+\omega_{22}\delta_{c2}+\omega_{23}\delta_{c3}\right)$

The weighting values can be adjusted as:

$\omega_{21}=-z_{1}^{\left(1\right)}$, $\omega_{22}=\omega_{22}-\mu_{1}\delta_{c2}o_{b}$, $\omega_{23}=\omega_{23}-\mu_{2}\delta_{c3}o_{b}$, $\omega_{11}=\omega_{11}+o_{a}t\delta_{b}$

where $\mu_{1}$ and $\mu_{2}$ are learning rates, which are defined previously.

The threshold value can be adjusted as:

$$\theta =\left(1+e^{-\omega_{11}t}\right)\left(\frac{\omega_{22}}{2}y_{t}^{1\left(1\right)}+\frac{\omega_{23}}{2}y_{t}^{2\left(1\right)}-z_{1}^{\left(1\right)}\right)$$

*Step 4:* Back to Step 2, re-adjust the weighting values and the threshold value until GNN is convergent.

GNN has a rapid convergence rate. Usually, the optimal weighting values and threshold value can be achieved after adjusted twice. After the GNN is convergent, it can be utilized to efficiently predict the corresponding position error, which is calculated by taking the inverse accumulated generation operation (IAGO) operation on ${\hat{z}}_{t}^{\left(1\right)}$.

$${\hat{z}}_{1}^{\left(0\right)}={\hat{z}}_{1}^{\left(1\right)}$$

$${\hat{z}}_{t}^{\left(0\right)}={\hat{z}}_{t}^{\left(1\right)}-{\hat{z}}_{t-1}^{\left(1\right)},t=2,\ldots ,N$$

## 5. Experiments and Results

### 5.1. Equipment and Road Trajectories

To evaluate the localization performance of the proposed solution, several experiments were conducted on a Chery TIGGO5 SUV (Chery Automobile Co., Ltd., Wuhu, China). Since the vehicle was equipped with ABS and ESP, the information about steering angle and forward speed could be directly obtained from the in-vehicle CAN bus. Besides, a low-cost NovAtel Superstar II GPS receiver (NovAtel, Calgary, AL, Canada) with 1 Hz rate and MEMSIC MEMS-based IMU VG440CA-200 sampled at 100 Hz were installed. The RISS data used in this research is from the one vertical gyroscope and two horizontal accelerometers of the full six-degree-of-freedom (6-DoF) IMU VG440CA-200. For the MEMS-based inertial sensors, the gyroscope has a bias stability of 10°/h and angle random walk of 4.5°/√h, while each accelerometer has bias stability of 1 mg and velocity random walk of 1 m/s/√h. The accuracies of other sensors (1σ) are 0.05 m/s and 3 m for the GPS velocity and position, 0.05 m/s for the wheel speed sensor, and 4° for the steering angle, respectively. Moreover, an accurate and reliable NovAtel SPAN-CPT system was used as a reference for quantitative comparison. The horizontal position accuracy of SPAN-CPT system was 0.01 m in absence of GPS outages and 0.02 m during 10 s outage.

Several road-test experiments were carried out along different trajectories using the setup described above. One of the trajectories was on the Fifth Ring Road in Beijing, which was a typical urban scenario with real GPS-denied environments in some parts. Besides, a series of typical driving maneuvers, such as lane-changes, accelerations and decelerations etc., were conducted according to actual driving conditions. It is worthwhile to mention here that, in this paper, that the position errors denote the horizontal Euclidean distance error between the estimated position and the corresponding reference, which is the main concern for land vehicle localization.

### 5.2. Test 1: Performance Evaluation of the Proposed Localization Solution in Trajectories 1

**Figure 5:**
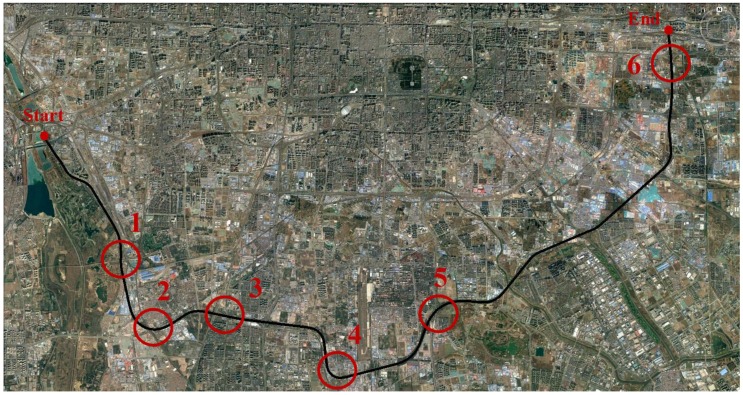
Trajectory 1 with GPS outages indicated.

The trajectory was shown in Figure 5. Straight portions and curves were considered when selecting outages in this trajectory. Since some periods of real GPS outages were shorter than 45 s, the selected outages were all extended to 45 s for convenient comparison.

In this test, the overall performance of the proposed localization solution was evaluated. As shown in Figure 1 and Figure 2, our proposed solution fuses the information from GPS, MEMS-RISS, and in-vehicle sensors utilizing IMM-UKF and compensates position errors utilizing GNN during GPS outages. Thus, the proposed solution is termed as IMM-UKF-GNN. In order to highlight the advantages of our proposed methodology, three other methods are also conducted for comparison: (1) General UKF without any compensation during GPS outages, termed as UKF; (2) IMM-UKF without any compensation during GPS outages, termed as IMM-UKF; (3) IMM-UKF with Radial Basis Function (RBF) compensation during GPS outages, termed as IMM-UKF-RBF. Note that both general UKF and IMM-UKF have the same motion model and measurement model described in Section 3. The difference is that the UKF method only has a constant Q matrix. In other words, the general UKF can be treated as one of the UKFs in the IMM-UKF. Both the UKF method and the IMM-UKF method can only execute the measurement update associated with in-vehicle sensors during GPS outages and without any further compensation. Since RBF has been widely regarded as the most remarkable ANN during the past decades [28], it is elected to compare with GNN in this paper. The RBF module was designed with the same inputs and outputs as GNN. Besides, the same 45 s sliding window was also utilized to train the RBF module. The learning procedures of both GNN and RBF continue as long as the GPS signal is available. In case of GPS outages, the trained RBF and GNN module are utilized to predict and compensate the position errors. In the absence of GPS outages, all the four methods can provide an accurate position output. Therefore, we focus on the comparisons of the performances among the four methods during GPS outages.

Table 1 and Table 2 give a quantitative comparison of the maximum and RMS position errors among the four methods described above during the six GPS outages, respectively. The highlighted columns correspond to the least errors achieved by the proposed localization solution. From the tables, it can be determined that the IMM-UKF method outperforms the UKF method. When GPS outages occur, the position errors will accumulate rapidly due to the uncertain noise of MEMS inertial sensors, the IMM-UKF can adapt to the uncertain noise and thus mitigating the error accumulation. On average, the IMM-UKF method achieves 9.9% and 13.6% improvements on maximum error and RMS error over the UKF method, respectively. However, both methods cannot ensure the localization accuracy and reliability during GPS outages.

The localization results also show that the methods with compensation can achieve much smaller errors than those without. Since the RBF and GNN can mimic the latest position errors, these errors can be removed from corresponding position components and thereby improving the localization accuracy during GPS outages. Furthermore, due to the advantages of GNN with respect to insufficient modeling information, the maximum error of the proposed IMM-UKF-GNN solution is 28.5% lower than that of the IMM-UKF-RBF method on average. When it comes to the RMS error, the proposed solution achieves a 28.1% lower value than IMM-UKF-RBF method.

**Figure 6:**
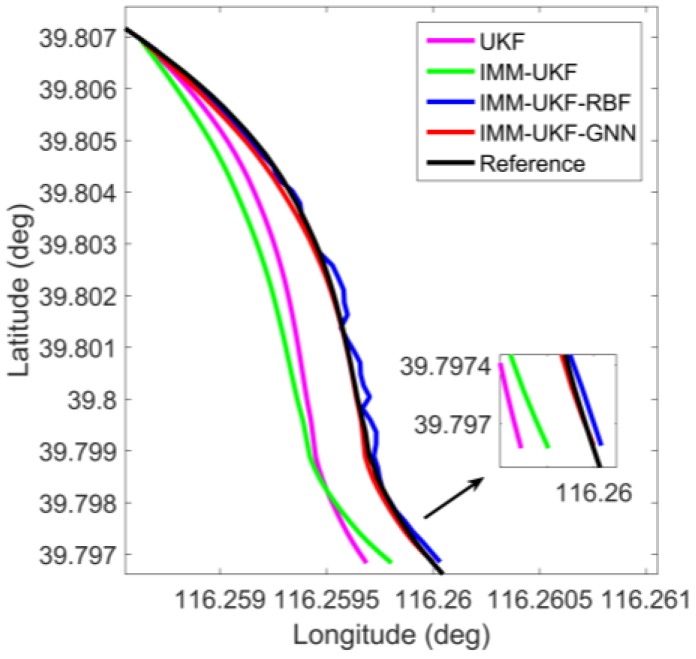
Localization results during GPS outage 1.

**Figure 7:**
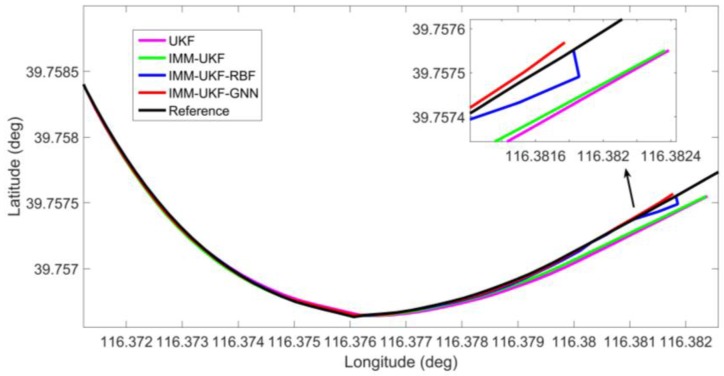
Localization results during GPS outage 4.

In order to directly show the localization results of different methods, three representative outages, i.e., outages 1, 3, and 4, were chosen to show the trajectories. Outages 1 and 4 correspond to the portion of the trajectory for the vehicle moving along curves, illustrated in Figure 6 and Figure 7. During outage 1, the percentage improvement of the proposed solution in maximum error is found to improve by 25.4%, 84.1%, and 85.1% against IMM-UKF-RBF method, IMM-UKF method and UKF method respectively, while the percentage improvement in RMS error is found to improve by 3.3%, 78.2%, and 81.5% respectively. For outage 4, the proposed solution effectively reduces the maximum error by 46.0%, 82.1%, and 82.7% against IMM-UKF-RBF method, IMM-UKF method, and UKF method, and reduces the RMS error by 51.5%, 80.2%, and 82.8%, respectively.

**Figure 8:**
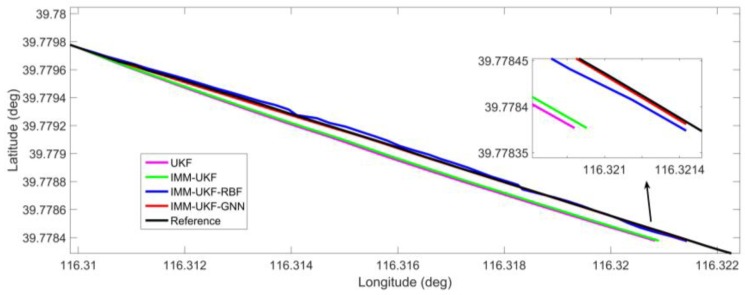
Localization results during GPS outage 3.

Figure 8 gives the localization results for outage 3, which belongs to a typical straight road. The percentage improvement of the proposed solution in maximum error is found to improve by 42.6%, 81.9%, and 84.1% against IMM-UKF-RBF method, IMM-UKF method and UKF method respectively and the percentage improvement in RMS error is 36.0%, 81.6%, and 85.9% respectively.

### 5.3. Test 2: Further Evaluation of the Proposed Localization Solution

In order to further test the adaption to uncertain noise of the proposed solution, we inserted biases into the inertial sensor data during the periods of GPS outages. The biases are modeled by the first-order Gauss-Markov process. The correlation time is defined as 100 s and the standard deviation of the white noise associated with the process is 10 mg for the accelerometers and 100°/h for the gyroscope. After inserting biases, the statistical properties of inertial sensor errors were dramatically changed during simulated outages, and the **Q** matrix should be updated correspondingly, which was not capable for the general UKF. Thus, the inaccurate **Q** matrix would cause performance degradation in the UKF method. However, the proposed IMM-UKF was envisioned to be adaptive to the inserted biases. Table 3 and Table 4 show the results of maximum and RMS position errors among the four methods during the six GPS outages after inserting biases. The proposed IMM-UKF-GNN solution can still achieve the best maximum and RMS position errors.

**Figure 9:**
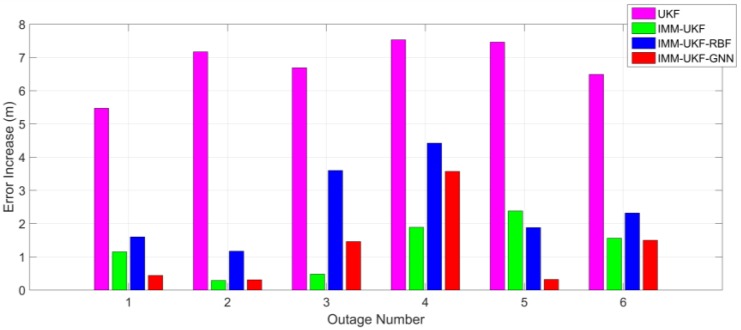
Increase of maximum error among the four methods after inserting biases.

**Figure 10:**
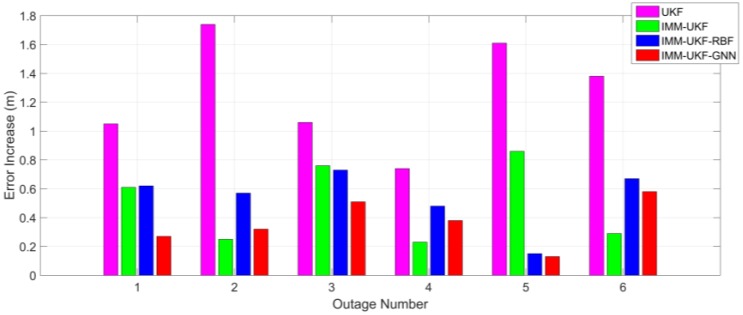
Increase of RMS error among the four methods after inserting biases.

Comparing Table 1 and Table 3, it can be determined that, after the statistical properties of inertial sensor errors are intentionally changed, the maximum error of the methods with IMM-UKF only increases 1.8 m on average while the increase of the UKF method is 6.8 m. The increase of the maximum position errors among the four methods is also shown in Figure 9. Besides, comparing Table 2 and Table 4 it can be found that the increase of RMS error is 0.47 m on average for the methods with IMM-UKF, while the increase is 1.26 m for the UKF method. The increase of the maximum position errors among the four methods is also depicted in Figure 10. Thus, it can be concluded that, when facing the same situation of uncertain inertial sensor noise, the IMM-UKF can achieved better performance than the general UKF.

## 6. Conclusions

This paper has presented a cost-effective vehicle localization solution, which can simultaneously address uncertain noises of MEMS inertial sensors and GPS outages. The proposed IMM-UKF fuses information from low-cost GPS, MEMS-RISS, and in-vehicle sensors. Three UKFs with different covariances are developed to cover a wide variation of inertial sensor noise. Then, an accurate estimation of vehicle positions can be obtained when GPS is available. Meanwhile, another IMM-UKF is developed to execute the measurement update associated with in-vehicle sensors. The difference between the state vector of the two IMM-UKFs are modeled by a GNN module. When GPS outages occur, the latest updated GNN module can predict and compensate position errors. Thus, the proposed solution can achieve accurate localization even without GPS observations.

The proposed localization solution has been successfully implemented and tested with real road-test trajectories. Through comparison with other three representative localization methods, it can be concluded that the research fulfills the basic aim of proposing a cost-effective vehicle localization solution which can maintain relatively good performance when facing uncertain inertial sensor noises and GPS outages simultaneously.

## Figures and Tables

**Table 1:** Maximum position errors during GPS outages.

Outage Number	Maximum Error (m)			
	UKF	IMM-UKF	IMM-UKF-RBF	IMM-UKF-GNN
1	26.32	24.62	5.24	**3.91**
2	55.20	46.94	16.21	**15.82**
3	64.62	56.76	17.92	**10.29**
4	53.64	51.74	17.20	**9.28**
5	59.98	51.76	12.28	**7.83**
6	26.13	23.83	11.66	**8.21**

**Table 2:** RMS position errors during GPS outages after inserting biases.

Outage Number	RMS Error (m)			
	UKF	IMM-UKF	IMM-UKF-RBF	IMM-UKF-GNN
1	6.31	5.42	1.22	**1.18**
2	13.84	12.96	7.69	**7.41**
3	21.41	16.43	4.72	**3.02**
4	16.54	14.34	5.86	**2.84**
5	18.41	14.23	2.81	**1.79**
6	7.95	7.81	3.71	**2.31**

**Table 3:** Maximum position errors during GPS outages after inserting biases.

Outage Number	Maximum Error (m)			
	UKF	IMM-UKF	IMM-UKF-RBF	IMM-UKF-GNN
1	31.79	25.77	6.84	**4.35**
2	62.37	47.23	17.38	**16.13**
3	71.31	57.20	21.52	**11.77**
4	61.17	53.63	21.62	**12.85**
5	67.44	54.14	14.16	**8.15**
6	32.62	25.39	13.98	**9.71**

**Table 4:** RMS position errors during GPS outages after inserting biases.

Outage Number	RMS Error (m)			
	UKF	IMM-UKF	IMM-UKF-RBF	IMM-UKF-GNN
1	7.36	6.03	1.84	**1.45**
2	15.58	13.21	8.26	**7.73**
3	22.47	17.19	5.45	**3.53**
4	17.28	14.57	6.34	**3.22**
5	20.02	15.09	2.66	**1.66**
6	9.33	8.10	4.38	**2.89**
